# Supplementary material for: Discovering Glioma Tissue through Its Biomarkers’ Detection in Blood by Raman Spectroscopy and Machine Learning
Source: Pharmaceutics. 2023 Jan 6;15(1):203. doi: 10.3390/pharmaceutics15010203 (PMC9862809; doi:10.3390/pharmaceutics15010203)
Supplement: Supplementary file 1 [file pharmaceutics-15-00203-s001.zip › pharmaceutics-2117731-supplementary.pdf]

pharmaceutics

MDPI

Supplementary Materials: Discovering Glioma Tissue through its Biomarkers' Detection in Blood by Raman Spectroscopy and Machine Learning

Denis Vrazhnov, Anna Mankova, Evgeny Stupak, Yury Kistenev, Alexander Shkurinov, and Olga Cherkasova

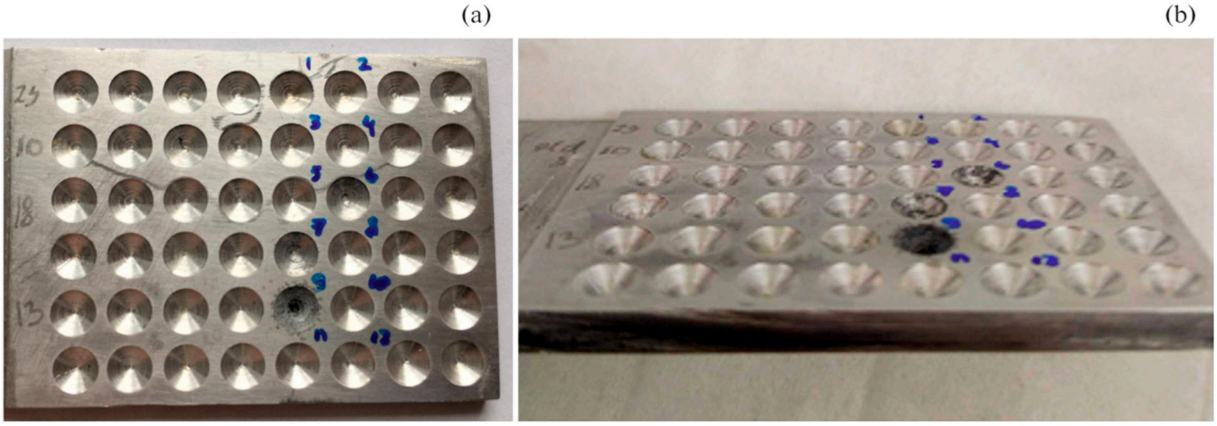

Figure S1. Special aluminum plate: (a) top view; (b) side view.

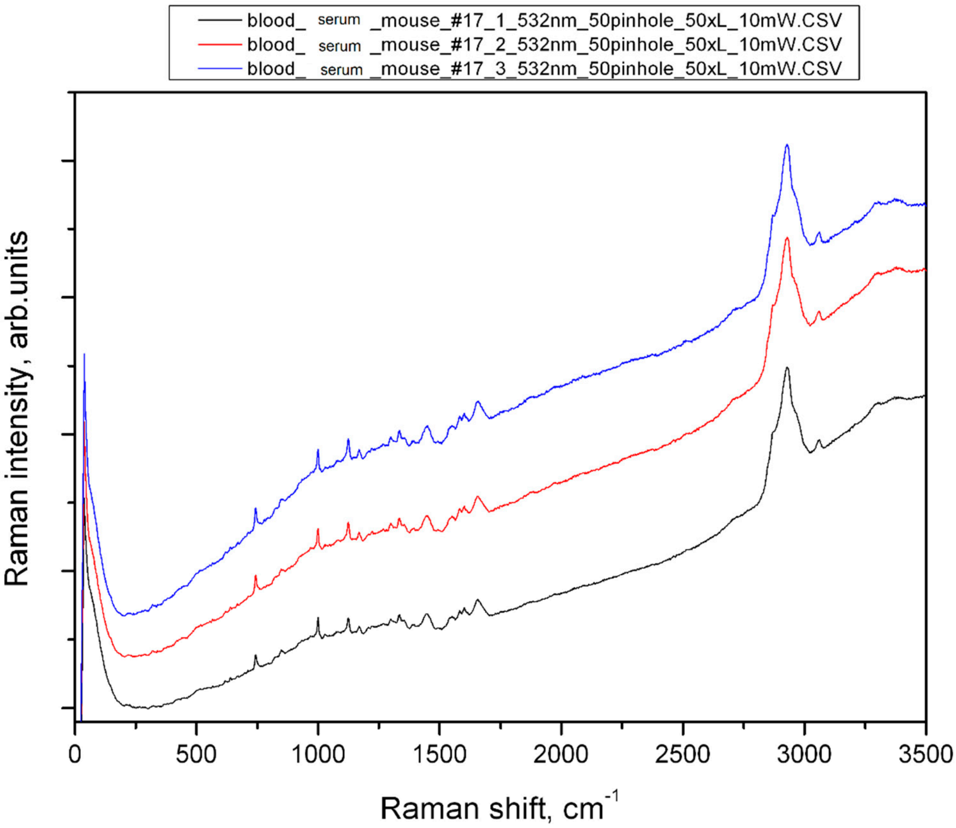

Figure S2. The three original Raman spectra of different parts of dried blood serum sample#17.

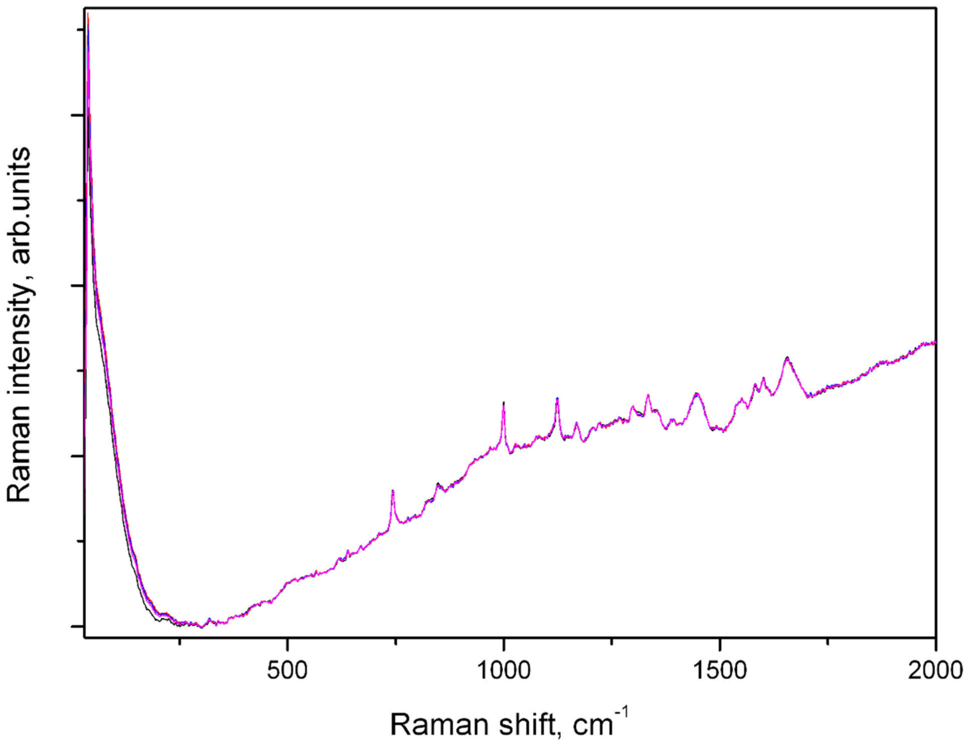

Figure S3. The average Raman spectrum of dried blood serum sample#17: gray line - spectrum #1; red line - spectrum #2; blue line - spectrum #3, magenta line - average spectrum from spectra 1-3.

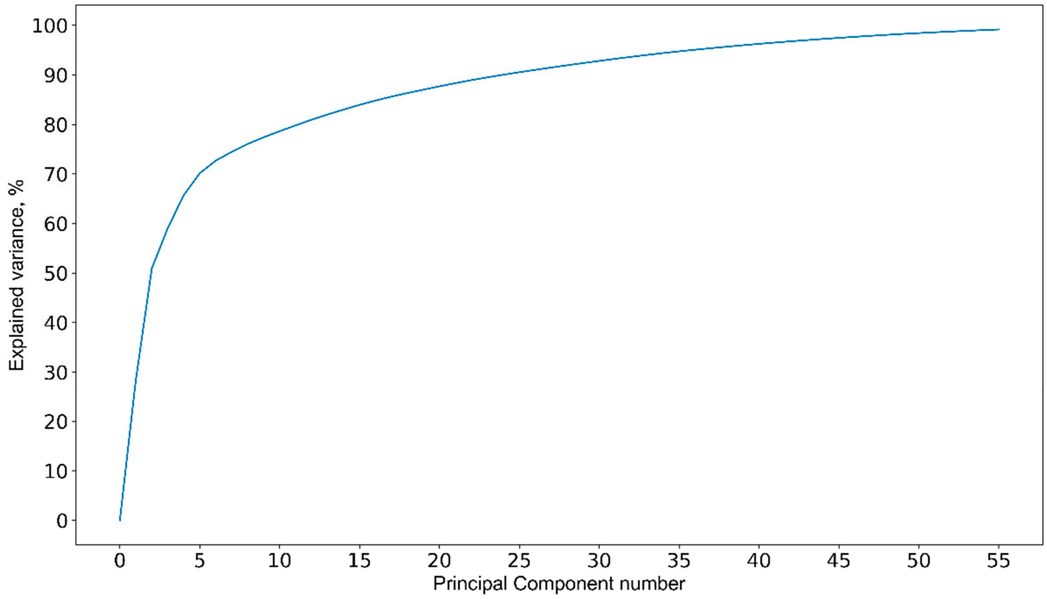

(a)

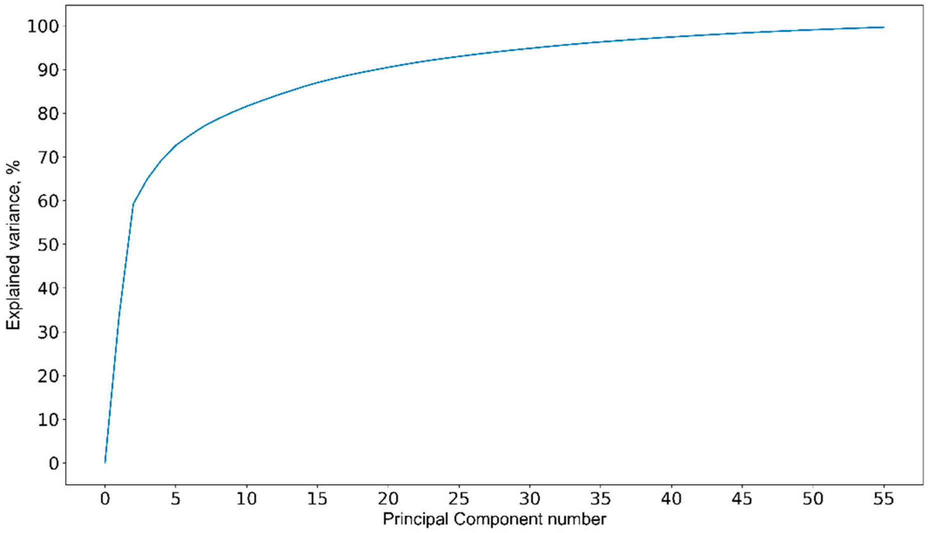

(b)

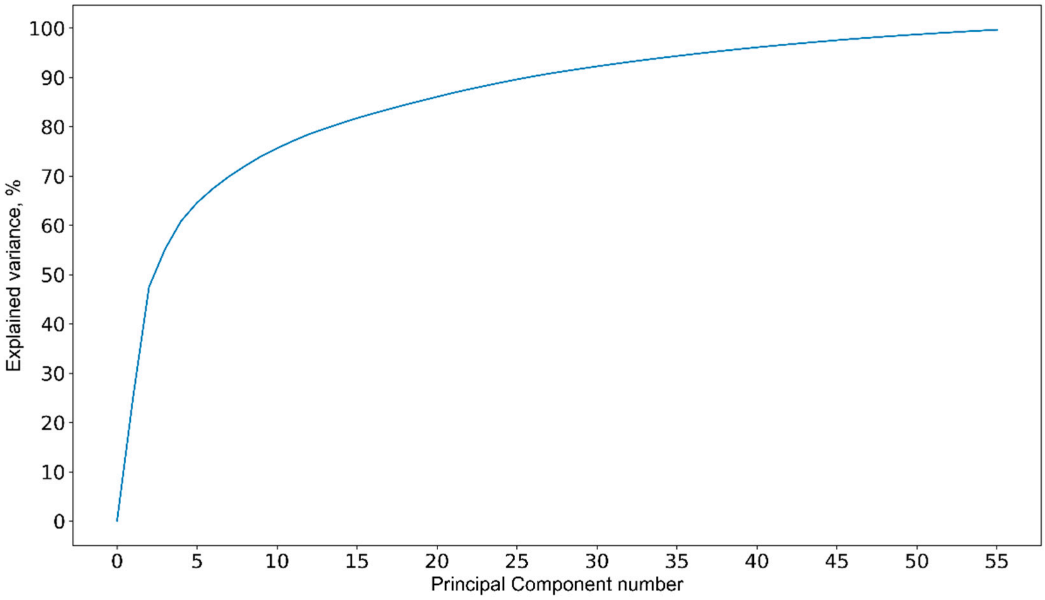

(c)

Figure S4. PCA explained variance in dependence on the number of principal components groups for 1st (a), 2nd (b), and 3rd (c) weeks of the experiment.

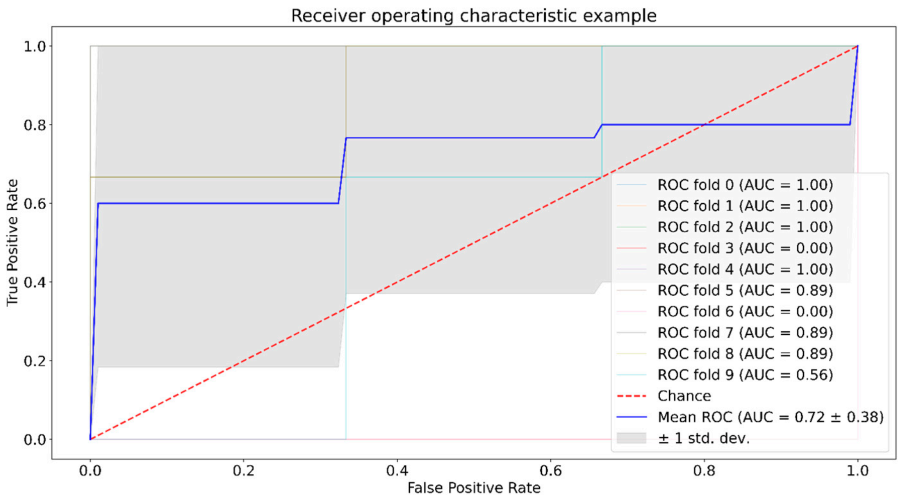

(a)

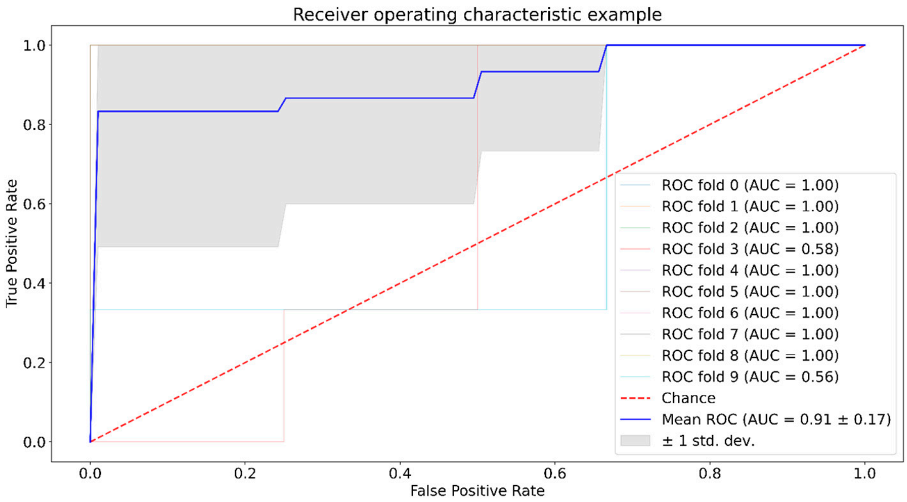

(b)

Figure S5. ROC-AUC analysis for SVM, 1st week no PCA (a) and with PCA (b).

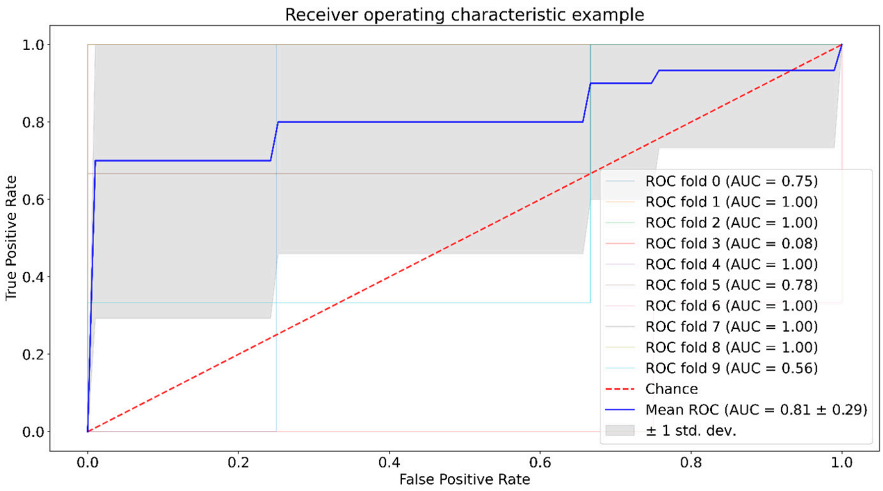

(a)

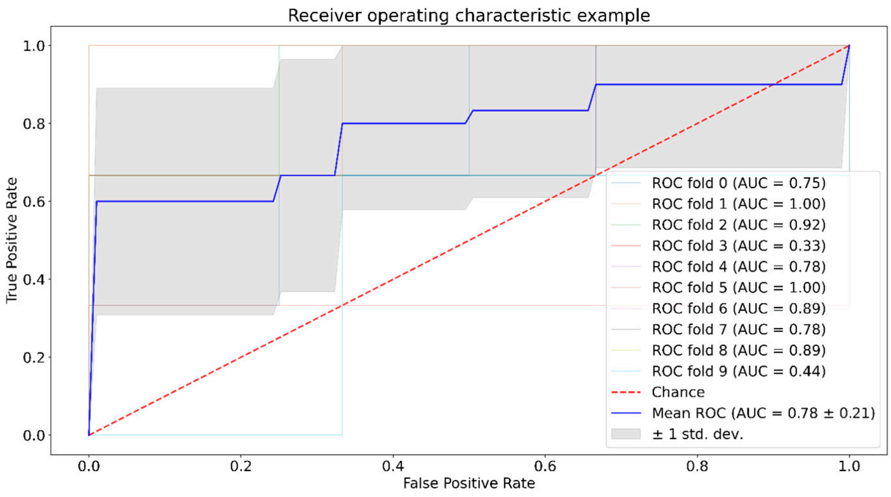

(b)

Figure S6. ROC-AUC analysis for RF, 1st week no PCA (a) and with PCA (b).

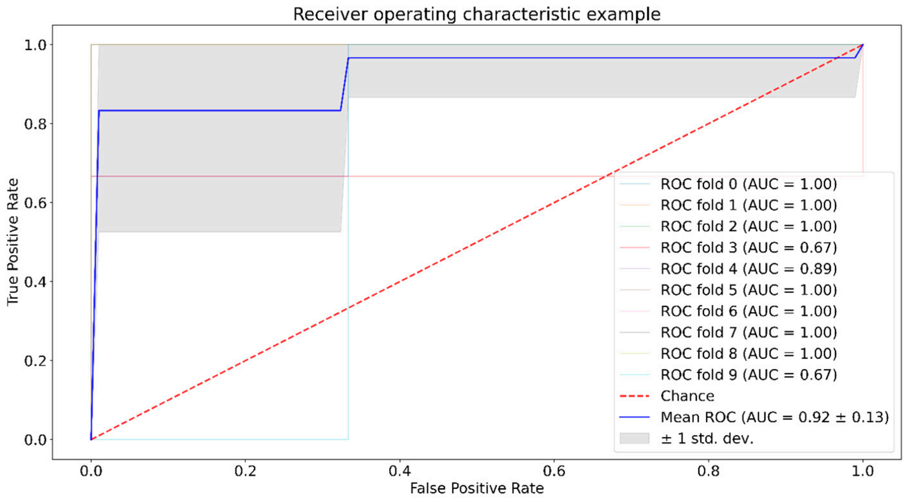

(a)

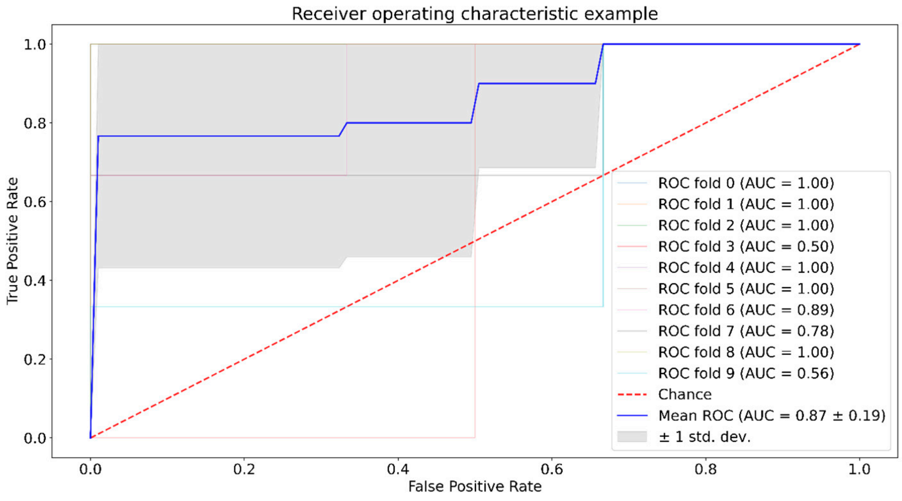

(b)

Figure S7. ROC-AUC analysis for XGBoost, 1st week no PCA (a) and with PCA (b).

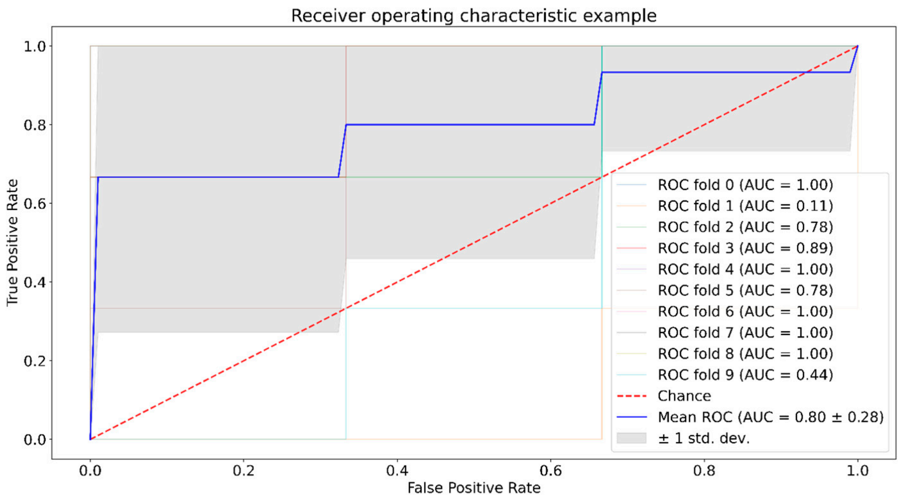

(a)

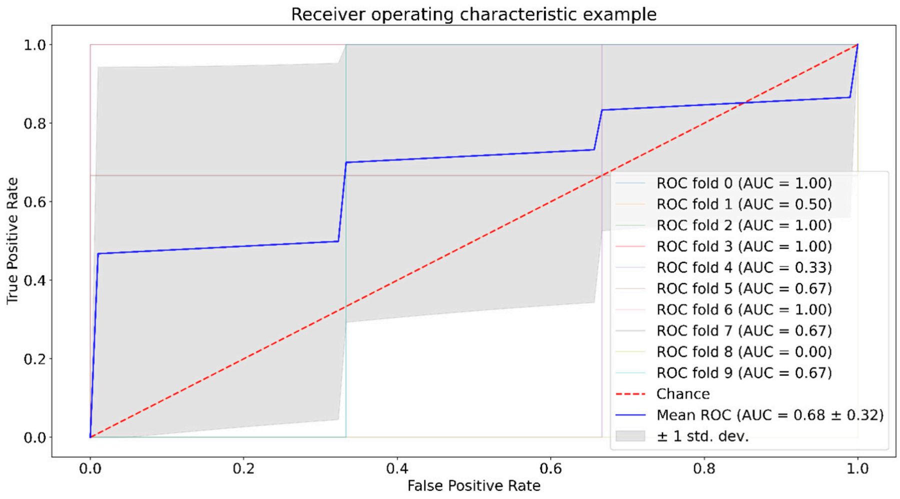

(b)

Figure S8. ROC-AUC analysis for SVM, 2nd week no PCA (a) and with PCA (b).

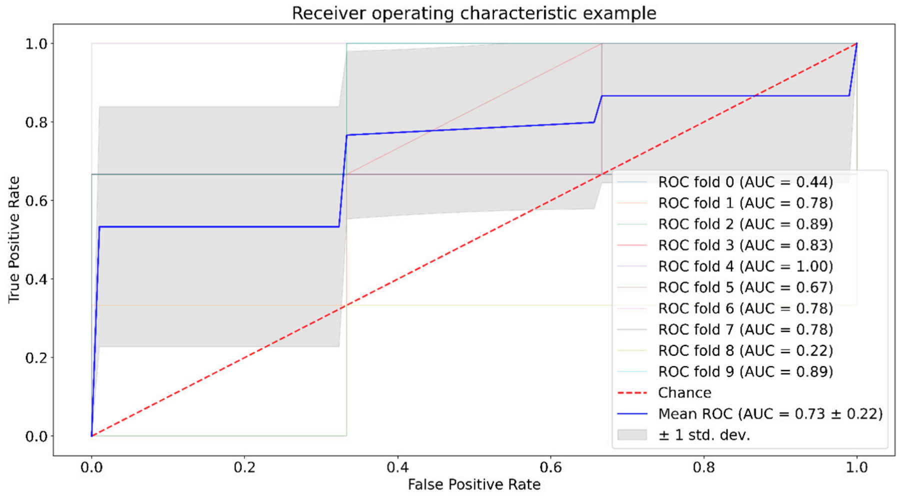

(a)

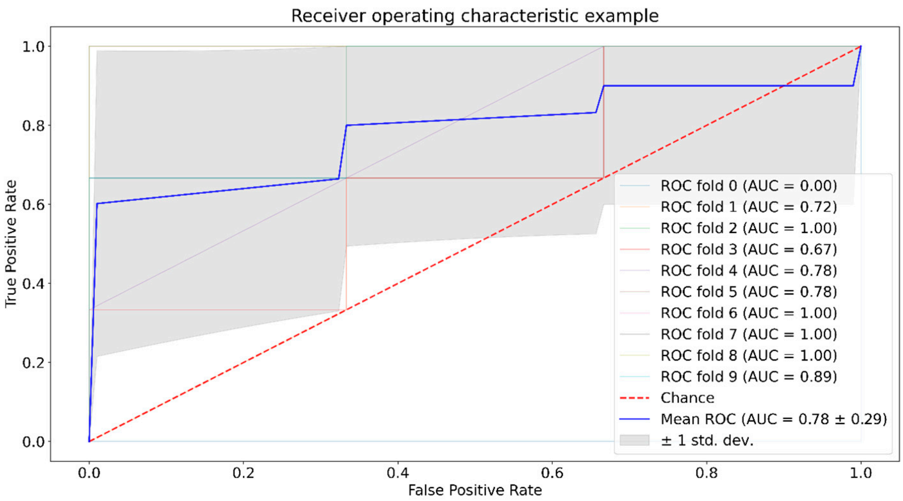

(b)

Figure S9. ROC-AUC analysis for RF, 2nd week no PCA (a) and with PCA (b).

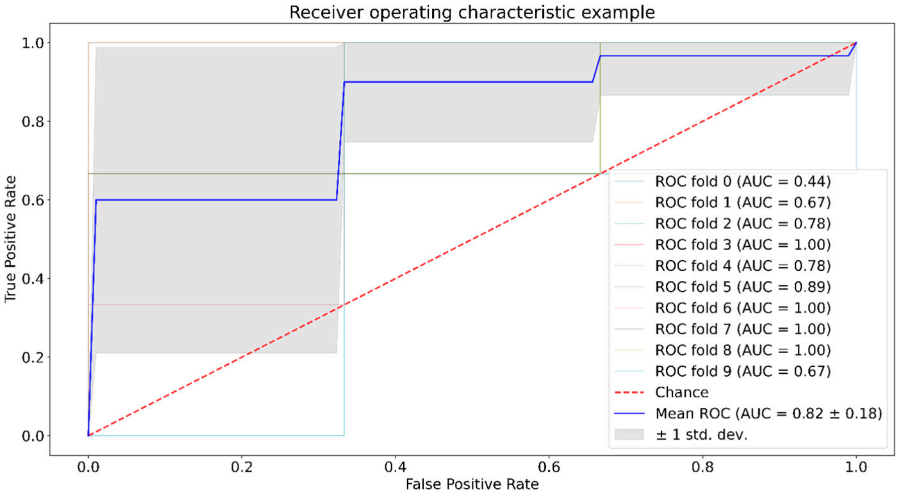

(a)

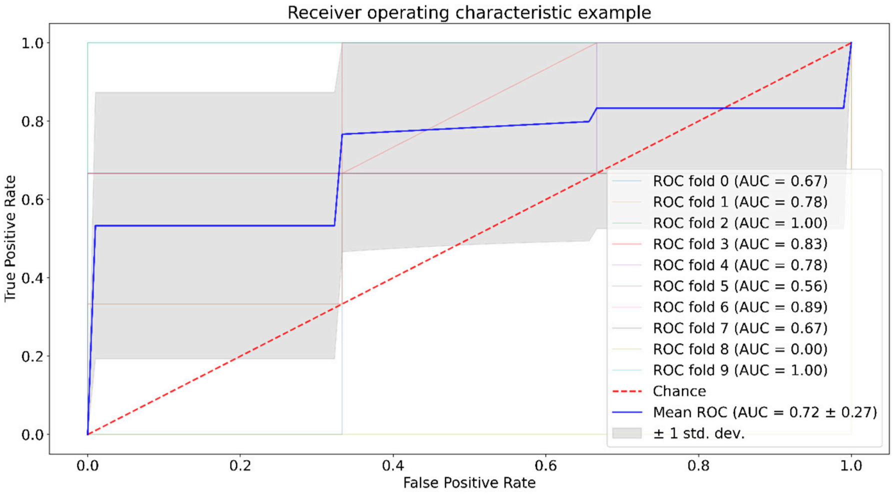

(b)

Figure S10. ROC-AUC analysis for XGBoost, 2nd week no PCA (a) and with PCA (b).

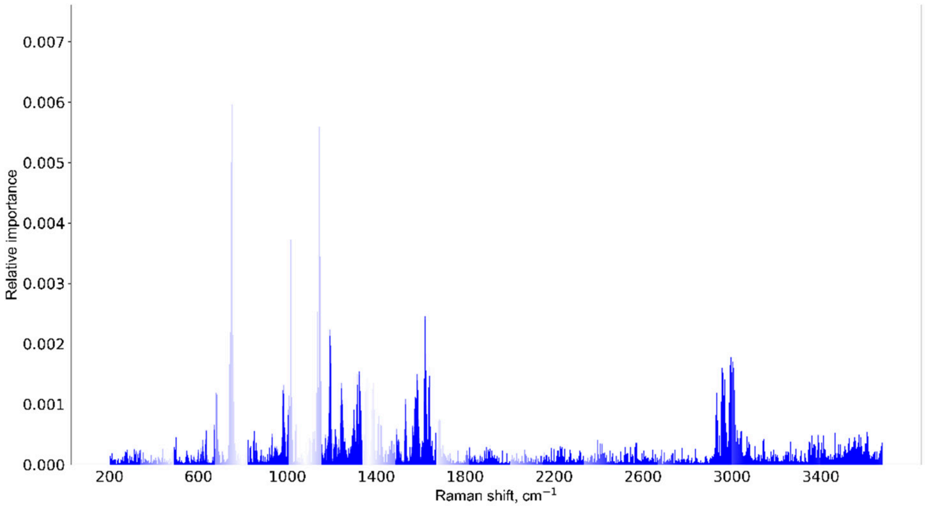
Relative importance
Raman shift, cm^{-1}

(a)

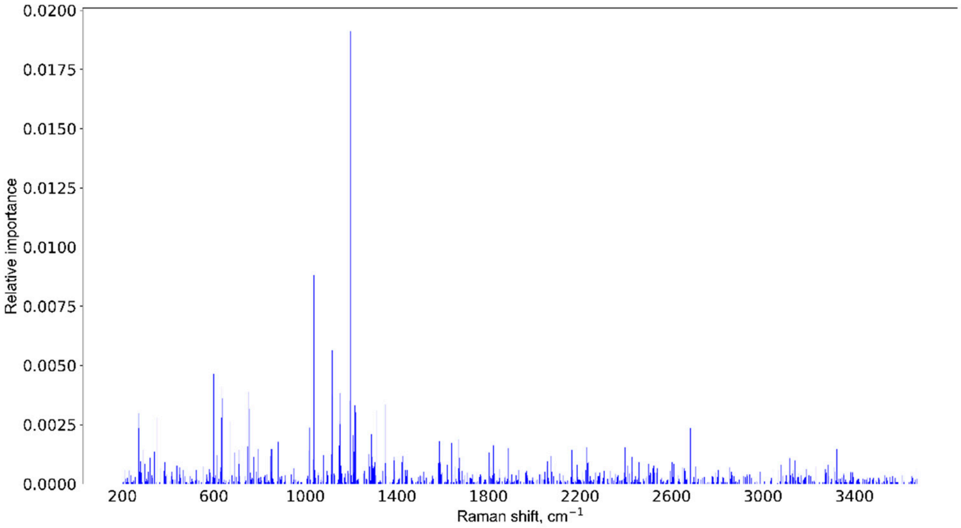
Relative importance
Raman shift, cm^{-1}

(b)

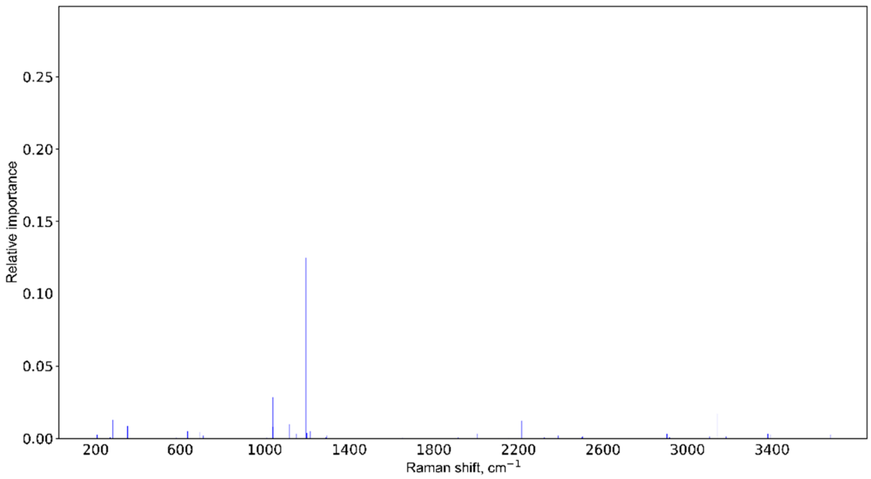
Relative importance
Raman shift, cm^{-1}

(c)

Figure S11. Informative feature selection for the first week of experiment for SVM (a), RF (b), and XGBoost (c) methods.

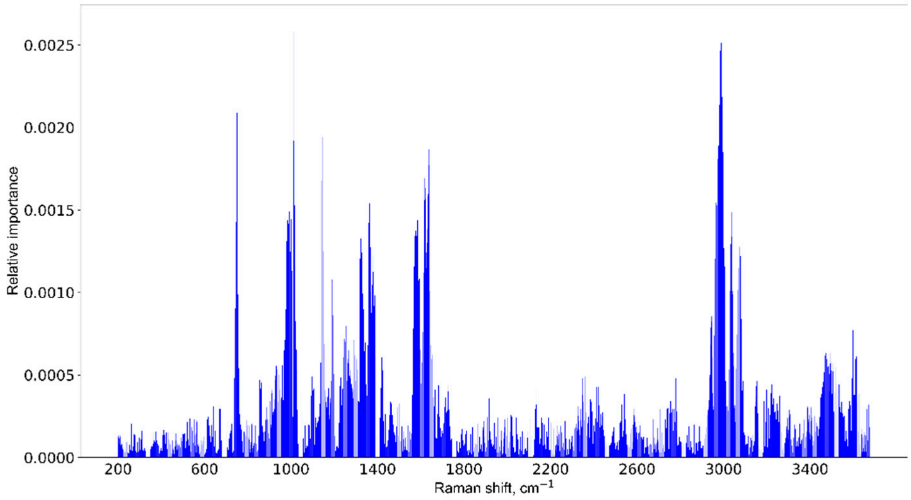

(a)

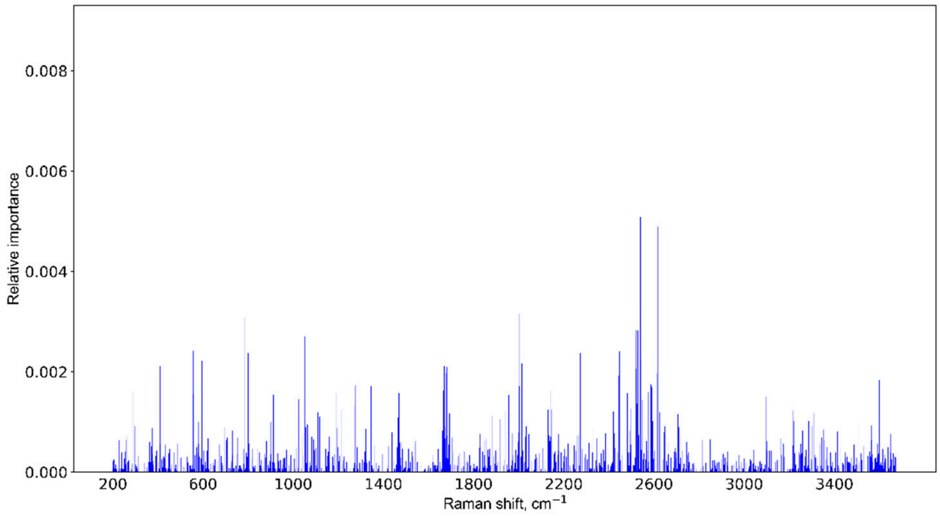

(b)

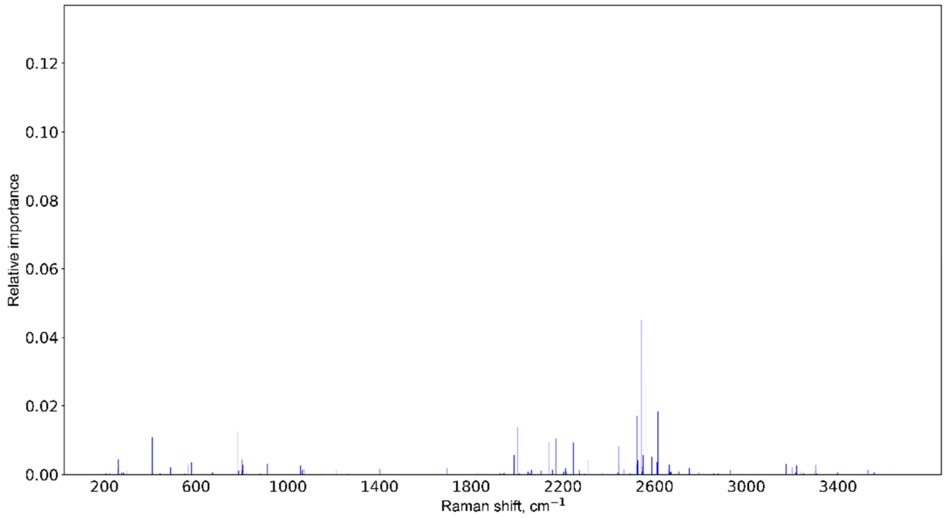

(c)

Figure S12. Informative feature selection for the second week of experiment for SVM (a), RF (b), and XGBoost (c) methods.
